# Evaluation of lordosis recovery after lumbar arthrodesis and its clinical impact

**DOI:** 10.1186/s41016-023-00333-4

**Published:** 2023-06-28

**Authors:** Gabriel Tchachoua Jiembou, Hermann Adonis Nda, Meleine Landry Konan

**Affiliations:** 1 Department of Neurosurgery, University Hospital of Toulouse, 31300 Toulouse, France; 2 Department of Neurosurgery, University Hospital Yopougon, 21 BP 632 Abidjan, Ivory Coast; 3 Training and Research Unit of Medical Sciences, University Felix Houphouet Boigny, 01 BPV 34 Abidjan, Ivory Coast

**Keywords:** Posterior lumbar arthrodesis, Sagittal alignment, Lumbar lordosis, Clinical outcomes

## Abstract

**Background:**

Posterior lumbar arthrodesis has become a widely used therapeutic option to correct sagittal imbalances in patients suffering from degenerative lumbar conditions. However, in western Africa, there is no study have reported long-term outcome of posterior lumbar arthrodesis. The aim of this study was to investigate the relationship between the restoration of adequate lordosis and the patient’s postoperative quality of life.

**Method:**

The study was retrospective. From January 2012 to December 2019, 80 patients who underwent posterior lumbar arthrodesis for lumbar degenerative diseases were included with a mean follow-up of 43.2 months. Mean age was 50.8 years (SD = 12.2). Preoperative and postoperative patients’ symptoms were assessed by the visual analog scale (VAS), Oswestry Disability Index (ODI), and 12-item Short Form (SF-12). Pre- and post-operative radiographic evaluation included lumbar lordosis measured (LLm), pelvic incidence (PI), sacral slope (SS), and pelvic stilt (PS). Theoretical lumbar lordosis (LLt) was defined by the following: LL = 0.54 × PI + 27.6. Data analysis was done using the statistical software “R.” The risk of error was 5% (*p* < 0.05).

**Result:**

The mean pelvic incidence was 57.23°. There was no statistically significant difference between preoperative and postoperative lumbar lordosis (*p* = 0.2567). There was no statistical difference between preoperative and postoperative PI-LL (*p* = 0.179). There was a statistically significant difference between the pre and postoperative clinical scores (*p < *0.001). Statistical analysis showed a correlation between recovery of lumbar lordosis and improvement in physical component of SF-12 (PCS) (*p* < 0.05) and lumbar and radicular VAS (*p* < 0.05) for the subgroup of narrow lumbar spine. There was a statistical relationship between the restoration of lumbar lordosis and improvement in PCS (*p* = 0.004) and VAS (*p* = 0.003) for the subgroup of isthmic lysis spondylolisthesis.

**Discussion:**

The root decompression performed in most patients could explain the clinical improvement regardless of recovery of lordosis. The failure to consider spinal parameters and sagittal balance of patients in the surgery could explain no restoration of lumbar lordosis. Our study had limitations inherent to its retrospective character such as the classic selection bias.

**Conclusion:**

Satisfactory correction of spinopelvic alignment may improve long-term clinical signs.

## Background

Chronic low back pain is a major worldwide problem. Prevalence in Africa is estimated between 28 and 74% (Louw et al. 2007 [1, 2]). These degenerative lumbar conditions can lead to long-term spinal imbalance. In Ivory Coast, its prevalence was estimated at 54.53% [1]. Although the first-line treatment is medical, failure of non-surgical management often requires posterior spinal arthrodesis aiming at the correction of sagittal imbalance. The importance of pelvic-spinal sagittal alignment in the management of lumbar degenerative disease has already been discussed [3, 4] that shows evidence linking postoperative sagittal alignment and improvement of patient quality of life [5]. Likewise, lumbar spine fusion performed in an unbalanced situation leads to the recurrence of disabling lumbar and radicular pain [3]. Anecdotally, Western Africans are deemed to have a more pronounced lumbar lordosis, and to the best of our knowledge, in this population, there is no outcome study on surgical restoration of the lumbar lordosis and the patient’s quality of life.

The aim of this study was to investigate the relationship between the restoration of adequate lordosis and the patient’s postoperative quality of life.

## Methods

This study was conducted in compliance with the Declaration of Helsinki and approved by the Ethics Committee of Yopougon Hospital University. Informed consent was signed by the patients or their legal representatives.

### Materials

Eighty (80) patients were included in this study: 38 were female (47.5%), and 42 were male (52.5%). The mean age was 50.8 years (SD = 12.2). All patients operated for lumbar degenerative disease by root release and posterior lumbar or lumbosacral arthrodesis at the Yopougon University Hospital between January 2012 and December 2019 were included. The patients with discogenic lomboradicular pain had postero-lateral interbody fusion. Twenty-six patients had a single spine level of arthrodesis, twenty-eight had two levels of arthrodesis, and twenty-nine had more than two levels of arthrodesis. Forty-one patients underwent arthrodesis of the lower lumbar spine (either L4-L5, L5-S1, or L4-S1). Three groups of pathologies were studied: narrow lumbar canal, spondylolisthesis, and disc herniation with signs of instability.

### Method

We performed a monocentric retrospective study of the clinical and radiological preoperative and postoperative characteristics of patients operated on for lumbar arthrodesis. The clinical characteristics of the patients were summarized by the ODI, the root and lumbar VAS, and the preoperative SF-12. Pelvic-spinal sagittal alignment parameters were studied on a full-spine radiograph in profile taking good views of the femoral heads. The radiographic image was digitized and integrated into the SURGIMAP SPINE® software. Pelvic incidence (PI), pelvic version (PT), and sacral slope (SS) were measured. Preoperative L1-S1 lumbar lordosis (LL) was measured by the Cobb method as well as L4-S1 lordosis. These were also measured after surgery. The average follow-up time was 43.2 months. Theoretical lumbar lordosis was defined by the Le Huec formula [6]: LL = 0.54 × PI + 27.6. Loss of lordosis was investigated by the difference between the theoretical lordosis and the measured preoperative and postoperative lordosis (LLt − LLm). The mismatch between PI and LLm was investigated through the mathematical operation PI-LLm. Lordosis was inadequate at pelvic incidence when the PI-LL difference > 10° [7].

### Statistical analysis

Data analysis was done using the statistical software “R”. The simple description of the sample was possible through the calculation of proportions and means. Chi-square tests were used to determine significance between categorical variables (*p* < 0.05). The risk of error was 5%.

Differences between the preoperative and postoperative clinical scores were analyzed with Student’s *T* test when the data followed a normal distribution and by the Wilcoxon rank test otherwise. Correlations between the clinical and radiographic parameters were performed using a Pearson correlation test. Subgroup analyses were performed according to pathology and PI value (PI < 56° and PI ≥ 56°).

## Results

The mean pelvic incidence was 57.23° with extremes ranging from 28° to 107°. Stratification of the PI according to the work of Barrey et al. [6] allowed us to note a class I PI in 18 patients (22.5%), class II in 38 patients (47.5%), and class III in 24 patients (30%).

The mean preoperative pelvic stilt (PS) was 24.6°. The mean postoperative pelvic stilt was 22.01°. There was no significant difference between the preoperative and postoperative pelvic stilt (*p* = 0.1482). Pelvic retroversion was found in 48 patients (60%) before surgery and pelvic anteversion in 2 patients (2.5%) before surgery. The pelvic stilt was normal in 30 patients (37.5%).

The preoperative lumbar lordosis (LL) was on average 39.84° ± 15.68°.

The mean difference between theoretical and preoperative lordosis (LLt − LLpreop) was 18.02°. The mean difference between the theoretical lordosis and the postoperative lordosis (LLt − LLpostop) was 15.13°. There was no statistically significant difference between preoperative and postoperative lumbar lordosis (LLt − LLm, *p* = 0.2567). There was no statistical difference between the preoperative and postoperative PI-LL (17.22° vs 13.76°; *p* = 0.179). The lumbar lordosis was not adapted to the pelvic incidence preoperatively and postoperatively. The preoperative clinical evaluation revealed an ODI score of 37.1, the lumbar VAS averaged 8.3, and the radicular VAS was 8.2. The preoperative MCS was 27.1, and the PCS was 18.5. The postoperative ODI was 9.3, VASL was 2.9, VASR was 2.3, MCS was 49.5, and PCS was 23. There was a statistically significant difference between the preoperative and postoperative clinical scores (*p < *0.001). The evolution of the clinical scores is summarized in Table 1.

**Table 1:** Outcomes according to clinical scores

	Preoperative	Last follow-up	Standard deviation	*P*
ODI	37.1	9.3	2.8	< 0.001
VAS.L	8.3	2.9	0.6	< 0.001
VAS.R	8.3	2.3	6	< 0.001
MCS	27.1	49.5	22	< 0.001
PCS	18.5	23	4	< 0.001

The mean preoperative sacral slope (SS) was 32.63°. The mean postoperative sacral slope (SS) was 35.22°. The upper arc of lordosis (L1S1 − L4S1) was not significantly improved (28.02° vs 27.19°, *p* = 0.6647). Table 2 summarizes the differences between the preoperative and postoperative spinal pelvic parameters at the last follow-up.

**Table 2:** Spinopelvic parameters in preoperative versus postoperative last follow-up

***Spinopelvic parameters***	***Preoperative***	Postoperative	*P*
LLt − LLm	18.02	15.13	*0.2567*
PI-LL	17.22	13.76	*0.1795*
Percentage of L4-S1 lordosis (%)	75.52	69.27	*0.141*
L1S1-L4S1 lordosis (°)	28.02	27.19	*0.6647*
Pelvic stilt	24.66	22.01	*0.1482*

**Fig. 1:**
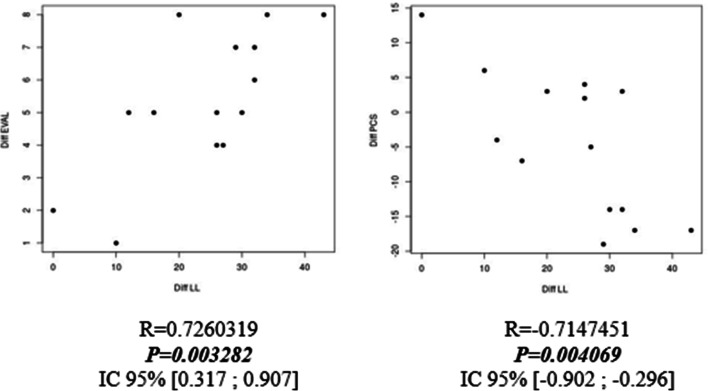
Correlation tests between the lordosis difference and EVAL Score (left); and PCS score (right) for the sub group of degenerative spondylolisthesis

**Fig. 2:**
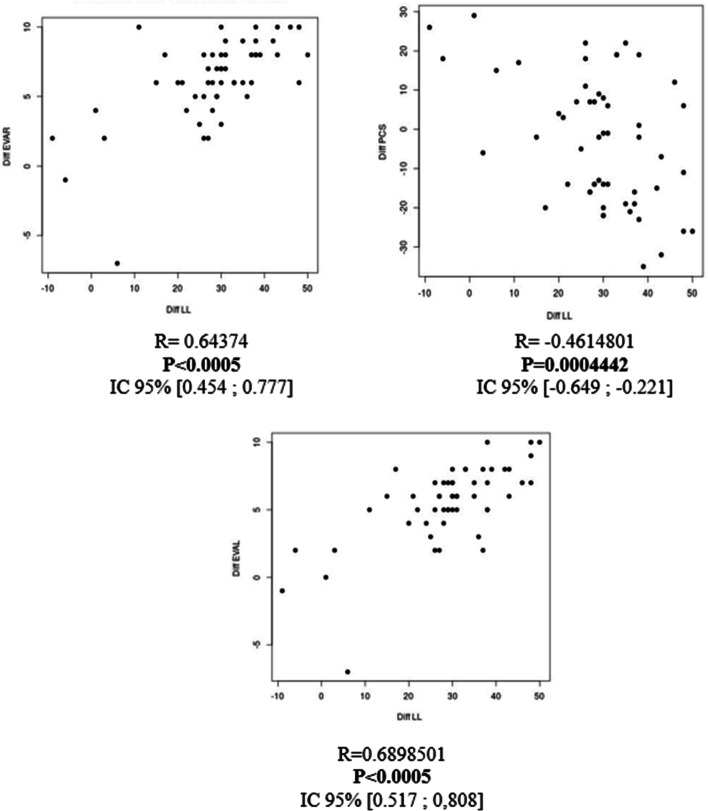
Correlation tests between of Lordosis difference and EVA R score (upper left); PCS score (upper right); and EVA L score (lower box) for the sub group of patients operated for narrow lumbar spine

Statistical analysis showed a statistical correlation between the recovery of lumbar lordosis and improvement in PCS (*p* < 0.05), VAS.L (*p* < 0.05), and VAS.R (*p* < 0.05) scores for the subgroup of narrow lumbar spine (Fig. 1). There was also a statistical relationship between the restoration of lumbar lordosis and improvement in PCS (*p* = 0.004) and VAS.L (*p* = 0.003) for the subgroup of isthmic lysis spondylolisthesis (Fig. 2).

Adjacent syndrome was the main complication found in 11 patients (13.75%) at the last follow-up.

## Discussion

The clinical condition of the patients was significantly improved at the last follow-up without any statistical link with the restoration of lordosis. This could be explained by the fact that most of the patients had a radicular release, which allowed significant improvement in patients with essentially radicular symptoms. Lazennec et al. [8] reported that failure to restore lumbar lordosis postoperatively was not always associated with residual pain symptoms. The preoperative lumbar lordosis and the lumbar lordosis at the last recoil were inadequate for the pelvic incidence. Lumbar lordosis was not significantly restored, which may be due to the failure to consider spinal parameters and sagittal balance of patients in the preoperative strategy and planning for lumbar arthrodesis. There was a statistical relationship between restoration of lumbar lordosis and improvement in clinical scores such as VAS and the physical component of the SF-12 functional score for the spondylolisthesis and narrow lumbar canal groups. These results are in line with those of Petit et al. [5] who also reported a statistical relationship between restoration of adequate L1S1 lumbar lordosis and improvement in clinical scores for the groups of patients operated on for degenerative spondylolisthesis.

The preoperative and postoperative pelvic version was high in our study: 24.6° and 22.01° respectively (compared to a mean value of 13° ± 7 found in the general population [9]. Le Huec J-C et al. reported that a high pelvic version postoperatively was correlated with a loss of lumbar lordosis [10]. Kim et al. [11] and Lazennec et al. [8] reported better clinical outcomes in patients with a significant improvement in pelvic version after arthrodesis. The persistence of pelvic retroversion postoperatively would indicate an arthrodesis on an unbalanced spine.

The upper arc of the lordosis remained high at the last recoil for patients with a low arthrodesis (L4-S1), and it was not significantly lower than in patients with a low arthrodesis. The upper arc of lordosis remained high at the last recoil for patients with a low arthrodesis (L4-S1), which could be a compensatory mechanism for the loss of lower segmental lordosis after arthrodesis. The high values of the upper arc of lordosis show that a defect in L4-S1 segmental lordosis could trigger compensatory mechanisms in the upper segments, resulting in increased muscle work and increased stress on the arthrodesis.

The rate of adjacent syndrome was 13.75% in our study with 3 repeat surgeries for arthrodesis extension. This rate is significantly higher than recent data in the literature [12]. Indeed, the incidence of adjacent syndrome increases with time; Sears et al. reported an incidence of 2.5% per year after arthrodesis [12]. This result could be explained by the failure to restore lumbar lordosis.

Djurasovic et al. showed that patients who developed an adjacent syndrome had a significantly lower postoperative LL and thus a high difference (LLt − LLm) [13]. Studies considering the PI showed that a postoperative LL-PI difference of more than 10° was a risk factor for the occurrence of AS [7, 14]. These data are explained by several biomechanical studies that find an increase in intervertebral and posterior column stresses in adjacent floors in case of lordosis defect [6, 15].

Our study had limitations inherent to its retrospective character such as the classic selection bias. Also, the short follow-up time does not allow us to draw any relevant conclusions. Nonetheless, this is the first study to address the outcome of lumbar spine fusion regarding the sagittal balance alignment in a West African population.

## Conclusion

The analysis of spinal-pelvic parameters is useful for planning spinal surgery strategy. It also allows a better understanding of the mechanisms of biomechanical decompensation after lumbar arthrodesis in the medium and long term. The lumbar pelvic spinal study and its compensatory mechanisms should be considered before any lumbar spinal surgery, especially when a fusion of the lower lumbar segments (L4-L5-S1) is envisaged. Restoration of lumbar lordosis adapted to pelvic incidence should be imperative in posterior lumbar fusions.

## Data Availability

Data is available upon request to the corresponding author.

## Abbreviations

AS: Adjacent syndrome
LL: Lumbar lordosis
LLm: Lumbar lordosis measured
LLt: Theoretical lumbar lordosis
LLpreop: Preoperative lumbar lordosis
LLpostop: Postoperative lumbar lordosis
MCS: Mental component of SF-12
ODI: Oswestry Disability Index
PCS: Physical component of SF-12
PI: Pelvic incidence
PS: Pelvic stilt
SD: Standard deviation
SF-12: 12-Item Short Form Health Survey
SS: Sacral slope
VAS: Visual analog scale
VAS.L: Lumbar visual analog scale
VAS.R: Radicular visual analog scale
